# Awareness and Knowledge of Cardiovascular Diseases and Its Risk Factors Among Women of Reproductive Age: A Scoping Review

**DOI:** 10.7759/cureus.49839

**Published:** 2023-12-02

**Authors:** Amirah Alshakarah, Deema Muriyah, Felwah Alsaghir, Rana Alanzi, Sara Almalki, Sarah Alsadan, Anwar B Alotaibi, Rasha Alshaalan, Tarfa Albrahim

**Affiliations:** 1 Department of Health Sciences and Clinical Nutrition, College of Health and Rehabilitation Sciences, Princess Nourah Bint Abdulrahman University, Riyadh, SAU; 2 Research, Johns Hopkins Aramco Healthcare, Dhahran, SAU

**Keywords:** women of reproductive age, cvd prevention, cardiovascular diseases, health literacy, cvd risk factor, cvd awareness

## Abstract

Cardiovascular disease (CVD) is the leading cause of morbidity and mortality in women. Despite the significant burden of CVD, knowledge and awareness of its risk factors among women are low. This review aimed to identify CVD awareness, knowledge, and risk factors for women of reproductive age from different countries and variables that influence health outcomes. Studies published from 2000 to 2023 were reviewed using PubMed, ScienceDirect, Elsevier, and electronic databases. A total of 50 studies were found, and 41 were excluded. The keywords used were "Knowledge of the risk factor of heart disease," "cardiac risk factors," "cardiovascular disease," "heart disease awareness," and "heart disease," combined with "women" and "reproductive age."

The review revealed significant gaps in the general awareness and knowledge of CVD risk factors among women of reproductive age. Many women were unaware of the symptoms and risk factors associated with CVD, leading to delayed diagnosis and poorer outcomes. Lack of education, low socioeconomic status, and limited access to healthcare were identified as contributing factors to this knowledge gap. Young women, particularly those with poor pregnancy outcomes, demonstrated limited awareness and perception of CVD risk. The findings suggest significant gaps in general awareness, knowledge of CVD risk, risk factors among women of reproductive age from different countries, and factors that influence their health outcomes. Targeted interventions are urgently needed to improve awareness and knowledge of CVD among women of reproductive age. Efforts should focus on educating women about CVD risk factors and prevention strategies before symptoms arise. Addressing socioeconomic and educational disparities is crucial to bridging the gap in awareness. By enhancing awareness and knowledge, women can be empowered to take preventive actions and reduce their risk of developing CVD. As a result, we recommend that there are significant opportunities to educate women about CVD risk and prevention before symptoms arise. In addition, there is a need to develop effective interventions to raise awareness among women of reproductive age to close the gap in awareness and knowledge of CVD.

## Introduction and background

According to the World Health Organization (WHO), cardiovascular diseases (CVDs) are the leading cause of death globally [1]. CVDs are not just major causes of death but are also becoming more important as causes of long-term impairment as medical care improves [2]. According to the WHO, 9.8% of all disability-adjusted life years will be affected by ischemic heart disease and cerebrovascular diseases by 2030 [2]. Many major clinical trials have shown that lifestyle strategies such as smoking cessation, good diet, physical exercise, weight control, and stress management can help prevent CVD field [3]. Among women, CVD is the leading cause of death worldwide, killing 8.6 million women each year [3].

Moreover, it has been recorded that 52% of women die before reaching a hospital after suffering a heart attack with pain in the center of the chest and sweating [2]. In contrast, non-specific symptoms include pain in the arms, left shoulder, jaw, and back; breathing difficulty; nausea; vomiting; lightheadedness; sleep disturbances; and fainting [2]. The reality of this circumstance is emphasized by the truth that most ladies are uninformed of the extent of their CVD [3]. The literature reflects women's misconception that CVD affects primarily males; due to this misinterpretation, women have delayed diagnosis, driving them to more awful results and raising disability for ladies with CVDs [4]. Women's awareness of risk factors, understanding of the disease, and state of consciousness are all areas of CVD prevention that have not received enough attention despite the literature developing evidence-based guidelines for identifying and treating CVD in women [4].

Data on young women's awareness and perception of CVD risk, particularly among those with poor pregnancy outcomes, is limited [5]. To establish primary prevention methods, studies in young adult populations are required [5]. Second, changes in study design and sample selection can explain some gender discrepancies [5]. Both men and women share some characteristics, and they are similar regarding physical inactivity, general CVD risk factors, and the onset of symptoms that differ between men and women [5]. Women, for example, often engage in less physical activity, while men smoke more [5]. Many women with CVD who passed away had no symptoms [6]. Menopausal women received more attention than women of childbearing age for CVD awareness [6]. However, comorbidities such as hypertension and gestational diabetes are more common in women of reproductive age due to pregnancy problems [6]. This review aims to determine how well women of reproductive age from various nations know CVD, risk factors, and factors that affect health outcomes [6].

Young women are our community's future leaders; they must maintain their health and have the required awareness and knowledge to protect themselves from chronic diseases that reduce their quality of life [4,5]. As a result, their health is a precise predictor of the future health demands and issues facing the next generation [4,5]. Women of reproductive age have more significant stress, responsibility, and pregnancy complications than any other age group [4,5]. The incidence of CVD continues to rise even though preventive measures can be done before the disease manifests [3]. Due to the cessation of estrogen in their bodies, women who have reached menopause have been the subject of numerous research fields [7-10]. The knowledge, the awareness, and the prevalence of CVD risk factors comprise three key categories [11]. Their search findings point to several articles, including low socioeconomic and educational levels and risk factors that women are unaware of, such as hypertension, smoking, gestational diabetes mellitus (GDM), and dyslipidemia [11]. Women of reproductive age are generally ignorant of the symptoms and risk factors associated with cardiac disease [11]. As a result, women do not adopt preventive actions to reduce their chances of acquiring CVD [11].

The aim of this scoping review was to assess the awareness and knowledge of CVD and its risk factors among women of reproductive age from different countries. The study aimed to identify the gaps in awareness and knowledge as well as the variables that influence health outcomes in this population. By examining the existing literature, the study sought to provide insights into the need for targeted interventions and strategies to improve awareness, knowledge, and preventive actions related to CVD among women of reproductive age.

## Review

Methodology

Literature Search and Study Selection

We conducted a comprehensive literature search from 2000 to 2002 using PubMed, ScienceDirect, Elsevier, and other electronic databases. The search strategy included the following keywords: "Knowledge of the risk factor of heart disease," "cardiac risk factors," "cardiovascular disease," "heart disease awareness," and "heart disease," combined with "women" and "reproductive age." The search yielded a total of 50 studies.

Evaluation of Sources

**Figure 1:**
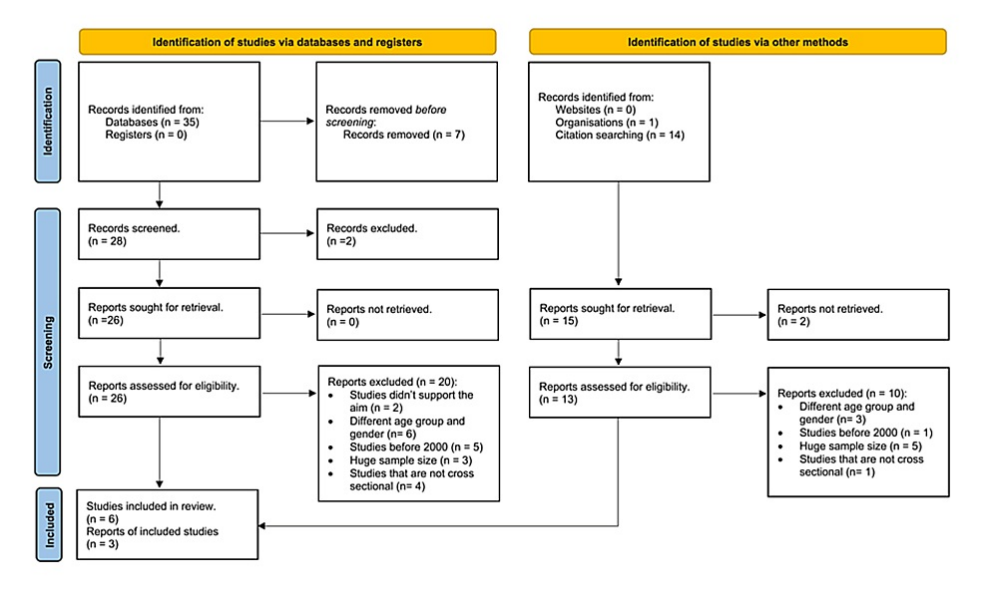
A framework for literature review methodology

Following the search, all identified citations were collected and uploaded to EndNote X9 (Clarivate Analytics, PA) [12]. We utilized the Preferred Reporting Items for Systematic Reviews and Meta-Analyses extension for scoping reviews (PRISMA-ScR) checklist for evaluating the individual sources of evidence [13]. This checklist provided a structured framework for assessing the quality and relevance of the included studies. Forty-one studies were excluded because of the following reasons: not cross-sectional, did not support this scoping review aim, before 2000, had a huge sample size, aimed at children and older people, did not target women, or focused on the treatment of CVD and the prevention of its risk factors rather than the awareness and knowledge (Figure 1). All nine collected studies were cross-sectional through call phone interviews, online surveys, questionnaires, physical examinations, and laboratories measuring weight, height, blood sugar, cholesterol levels, and body mass index (BMI) as shown in Figure 1.

Results

Awareness of CVDs

Irani et al. reported a cross-sectional cohort study about awareness of non-communicable diseases (NCDs) in women [14]. NCD refers to chronic conditions that cannot be transmitted between people, including CVD, which represents the greatest burden of disease that causes CVD [14]. This study targeted Swiss women aged 18 and older who spoke German or French [14]. Illiteracy and lack of Internet access were excluded. About 61.1% of participants had never heard of the term “non-communicable disease” before; but when differentiating between moderate (< 20 points) and high (≥ 20 points) NCD awareness levels, 42.1% fell into the first category [14]. Language origin, education, the significance of health status, and having children significantly impacted NCD awareness levels [14]. Accordingly, NCD awareness level was significantly more likely to be high in French-speaking, highly-educated mothers to whom health was important [14]. Sources of information were mainly environment (85.5%), media (newspaper 63.8%, TV 54.8%), and rarely physicians (21.3%) [14].

Another study was conducted by Ramachandran et al. in 2016 aiming to investigate awareness, knowledge, healthy lifestyle, and their associations with coronary heart disease (CHD) among working women in Singapore [4]. Among 200 women, a non-probability quota sample comprised 50 academic group participants, 80 administration group participants, and 70 ad-hoc group participants [4]. Using a self-administered questionnaire that included the Heart Disease Facts Questionnaire-2 (HDFQ-2), they demonstrated that 47% of women are aware that CVDs are the greatest cause of mortality among women [4]. A national survey was conducted by McDonnell et al. of Canadian women aged 25 and older about their heart health. A total of 1654 responses were collected, comprising 208 online and 1446 telephone responses from a randomly selected sample [11]; 12.5% of people responded [11]. The study compared women's perceptions of their heart disease risk to their self-reported risk status [11]. Education and higher household income increased awareness of some symptoms [11]. Women under 45 years old were less likely to recognize any possible signs [11]. Women above 55 years were more likely to identify the most frequently mentioned symptoms [11]. In addition, awareness of many of the symptoms of heart disease in women was associated with knowing someone with heart disease [11].

A descriptive study was conducted by Khan et al. using a survey instrument adapted from the American Heart Association National Survey with a sample of 676 Emirati women between 18 and 55 years who completed the questionnaire [2]. A total of 97.3% of participants were within the 18-45-year-old age group, with only 2.7% being 46-55 years old; there was a sign that fewer women from the older age group perceived themselves to lack awareness of heart disease compared to the younger women [11]. The study viewed low awareness of heart disease and associated risk factors in Emirati women; only 19.4% of participants were aware of heart disease [2]. The Internet was considered a key source of information for health-related matters among 67.2% of Emirati women; friends and family were the primary informants for 49.3% of the participants and television for 36.1% of the participants. Overall, only 17.8% of participants specified magazines as a source of information [2].

Knowledge of CVDs

According to a cross-sectional study conducted by Beussink-Nelson et al., among 714 women who had a recent live birth and a mean age of 34, 25% had adverse pregnancy outcomes (APOs) [5]. The study evaluated young women's knowledge and perception of CVD risk and whether these characteristics differed according to APO experience [5]. This study was conducted by a self-administered online survey adapted from the American Heart Association Survey of Women's CVD Awareness [5]. They still demonstrated gaps in young women's knowledge of CVD risk, particularly after an APO [5]. Mosca et al. estimated American women's contemporary awareness, knowledge, and perceptions of CVD risk [15]. A telephone survey of a random sample of white, black, and Hispanic women was undertaken in 2003, and the findings were compared to similar surveys done in 2000 and 1997 [15]. White women had the most effective awareness rate in each survey year; black women's knowledge increased dramatically from 1997 and 2000 to 2003, while Hispanic women's rates did not change much [15].

Prevalence of Risk Factors of CVD

A study was done by Kalaf et al. among (833) Young women in Al-Qassim, Saudi Arabia, aged between 20 and 40 years, to assess the level of risk for CVDs using questionnaire-based information as well as a measurement of height, weight, blood pressure, and blood glucose [6]. Only 15% of the samples were free of risk factors; the majority had either one (57.5%) or two (20.8%) risk factors [6]. Moreover, 6.7% were considered at high risk with three or more risk factors [6]. The most common risk factors were physical inactivity (74%), overweight, and obesity (25%-29%), respectively [6]. The main finding of this study was that the majority of young Saudi women are obese and physically inactive, which statistically significantly increases their risk for CVD [6]. On the other hand, hypertension, hyperlipidemia, and type 2 diabetes had lower prevalence [6]. Magro López et al. conducted a study in Biscay (northern Spain) to estimate the prevalence of principal cardiovascular risk factors among females aged between 16 and 65 years [16]. A total of 1317 women were included in the study, and for each participant, they recorded the following parameters: weight and height, physical activity, smoking, blood pressure, glycemia, total cholesterol, triglycerides, high-density lipoprotein (HDL) cholesterol, and low-density lipoprotein (LDL) cholesterol [16]. Regarding physical activity, 31.9% of the women had a sedentary lifestyle, and 48.4% did not exercise during their leisure time [16]. They found an average BMI of 24.9 ± 4.6 kg/m^2^, and 42.4% of the women were overweight [16]. Smoking prevalence was 31.9%, hyperglycemia was 3.3%, and hypertension was 26.7%, with a cut-off value of more or equal to 140/90 mmHg [16]. Total cholesterol values were more than 240 mg/dl in 26.2%, triglyceride levels were more than 200 mg/dl in 2.6%, LDL cholesterol was more than 160 mg/dl in 26.8%, and HDL cholesterol values were less than 45 mg/dl in 12.2% [16]. They showed that these risk factors increased with age and noticed that smoking had been seen as socially acceptable and that there was a lack of awareness of its harmful effects, which can lead to an increase in the number of young women smokers [16].

Awareness of Risk Factors of CVD

The Berlin Women's Risk Assessment (BEFRI), a randomized cross-sectional study by Oertelt-Prigione et al., included a randomized sample of urban women aged 25-74 years, of whom 1,066 completed a standardized questionnaire and participated in a comprehensive clinical evaluation [3]. Women underestimate the role of CVD as a cause of death, but little information is available on women's subjective risk perceptions [3]. Only 41.35% of all participants correctly classified their cardiovascular risk, and 48.65% had a combination of unemployment and other social risk factors (low income, low education, simple job, living alone, and having children) [3]. The socioeconomic factors of fewer than half of the women in the study population correctly assessed their cardiovascular risk, which was also underestimated [3]. This study found that age was the strongest predictor of risk underestimation in urban women and the least subjectively recognized cardiovascular risk factor, according to Beussink-Nelson et al. The same Chicago study mentioned earlier found that the proportion of women who accurately identified traditional CVD risk factors and preventive activities was similar between groups [5]. Obesity, hypertension, and smoking were properly identified as CVD risk factors by nearly all individuals (>96%) [5]. Most respondents identified low cholesterol (>70%) and regular exercise (>94%) as risk-lowering factors. In comparison, a minority (46%) identified breastfeeding as a factor linked with a lower risk of CVD [5].

The studies in Table 1 used surveys and questionnaires such as online self-administered surveys, standardized questionnaires, clinical examinations, and interviews through cell phones. The reason could be its simplicity of administering and scoring and relatively quick completion time. Studies were collected to illustrate that the age groups of females involved in the study were in their reproductive age. A study in Saudi Arabia by Kalaf et al. showed that groups with high risk for CVD accounted for 6.7%, having three or more risk factors, while 15% are free of CVD risk factors. The most common risk factors were obesity and low physical activity. A study in Spain by Magro López et al. includes smoking as a risk factor; in contrast, the previous research from Saudi Arabia informed that men are more likely to smoke than women in the region, so it was not considered a risk factor among Saudi women [5,13]. A comparison between these studies has been conducted to highlight that highly-educated mothers and females, in general, were more aware of heart disease and associated risk factors than secondary education or less. However, a study in Berline by Oertelt-Prigione et al. identified that 41.35% of participants correctly classified their cardiovascular risk, while 48.65% underestimated it [3].

**Table 1:** The level of awareness, knowledge of CVD, and its risk factors among women of reproductive age on a worldwide platform ^a^ BMI: Body mass index. ^b^ CVD: Cardiovascular diseases. ^c^ HDFQ-2: Heart Disease Fact Questionnaire-2. ^d^ AHAS: American Heart Association survey. ^e^ HDFQ: Heart Disease Fact Questionnaire. ^f ^REDCap is a secure web application for building and managing online surveys and databases. ^g^ NCD: Non-communicable diseases.

Year of publication	Country	Authors	Population	Age group (years)	Sample size	Method	Results
2003	Spain, Biscay	López et al. [16]	Female	16-65	1317	Self-administered questionnaire	The prevalence of smoking was 31.9%. The mean BMI^a^ was 24.9 ± 4.6 kg/m^2^, and 42.4% of the women were overweight. The prevalence of hypertension was 13.1%, which increased to 26.7% when a cut-off value of ≥140/90 mmHg was used. The prevalence of hyperglycemia was 3.3%.
2004	United States	Mosca et al. [15]	Female	25 or older	1024	The telephone survey of a nationally representative random sample of women was evaluated using a standard interviewer-assisted questionnaire.	46% of respondents spontaneously identified heart disease as the leading cause of death in women, up from 30% of women (<45 years old) with lower awareness of heart disease as their leading cause of death than white and older women.
2014	Canada	McDonnell et al. [11]	Female	25 or older	1654	Random-digit-dial landline/cell phone	High scores were twice as likely among university-educated women (30%) than in those reporting secondary education or less (15%).
2015	Berlin	Oertelt-Prigione et al. [3]	Urban female	25-74	1066	Standardized questionnaire clinical examination	Only 41.35% of all the participants correctly classified their CVD^b^ risk, while 48.65% underestimated it.
2016	Saudi Arabia	Kalaf et al. [6]	Young female	20-40	833	Cross-sectional	Out of all the participants, 15% did not have any risk factors. The majority, which accounted for 57.5%, had one risk factor, while 20.8% had two risk factors. Moreover, a small percentage of participants (6.7%) were categorized as high risk due to having three or more risk factors.
2016	Singapore	Ramachandran et al. [4]	Working woman	21-63	200	A self-administered questionnaire, including HDFQ-2^c^	47% of the participants were aware of CVD^b^.
2017	United Arab Emirates (UAE)	Khan and Ali [2]	Emirati women	18-55	676	Survey instrument adapted from the AHAS^d^	The research findings indicated that Emirati women have a low understanding of heart disease and the factors that contribute to it. Surprisingly, 19.4% of the participants showed awareness of heart diseases.
2022	Chicago	Beussink-Nelson et al. [5]	Woman with a recent live birth	Mean age of 34	714	Self-administered online survey adapted from the AHAS^d^ of women's CVD^b^ awareness HDFQ^e^, REDCap^f^.	Knowledge of CVD^b^ and its risk factors: 62.1% APO group; 61.7% non-APO. Obesity, HT, smoking > 96%, PA >94%, low cholesterol > 70%.
2022	Western countries	Irani et al. [14]	Women in Switzerland	18 or older	221	Self-administered questionnaire (online survey)	The research discovered that mothers with high levels of education had a greater understanding of non-communicable diseases (NCDs)^g^.

Discussion

In this review, we identified low levels of CVD awareness and knowledge. Many women who were categorized as having a high risk of heart disease due to their lifestyle habits or medical history significantly underestimated their risk. Although many women believe that their health is primarily their responsibility, they have a poor understanding of heart disease and are unaware of their risk status. In addition, social risk factors were found to be significant determinants of underestimation. Other socioeconomic risk factors, such as poor education, low income, living alone, having children, pregnancy, and a lack of integrated insurance coverage, were only related to underestimation when combined in groups of three or more. These findings indicate significant gaps in women's knowledge and understanding of heart disease symptoms and the most critical risk factors for heart disease, even though there are well-established methods to lower risk.

Moreover, that finding is constant, with studies suggesting that the mortality rate was somewhat lower if the woman had just heard of CVD before being affected [14]. In most of the studies reviewed, awareness of CVD risk factors was shown to be generally inadequate [2,3,6,11,12,14]. Despite this, most young women are obese and physically inactive, considerably increasing their CVD risk. The prevalence of the other risk factors, hypertension, hyperlipidemia, and type 2 diabetes was lower. Physical inactivity has been identified as one of the greater risk factors among women, which may be explained by the fact that most of the respondents in this group were students who would spend a significant amount of time sitting down [2,3,6,11,12,14].

Even though smoking has been recognized as a risk factor for CVD in several studies due to its social acceptance and a lack of awareness of its dangers, which has led to an increase in the number of young women who smoke, one study found no smokers among its sample of young women [2,3,6,11,14,16]. Age is also thought to play a role in CVD because a study revealed that women above 30 years old are at a higher risk of CVD. Many women did not know the levels of their risk factors, such as cholesterol, indicating a lack of understanding of CVD risk factors among young women. Future public health efforts should prioritize disseminating more precise and accurate CVD information. Moreover, healthcare providers and nurses should consider the population of women at risk and those who are less likely to engage in preventive health screening and healthy lifestyle behaviors by providing personalized education.

Furthermore, future research must expand outreach to include gender and age-specific awareness of heart disease risks and symptoms. It is also necessary to highlight the potentially modifiable barriers to seeking health care, which should be overcome to reduce morbidity and mortality due to heart disease among women of reproductive age. Authorities will need to develop better prevention and treatment strategies and intervene earlier to reduce the risk of CVD among young women.

The current study showed strengths through its comprehensive literature review, which conducted a thorough examination of awareness and knowledge concerning CVD among women of reproductive age across various countries. A notable accomplishment of the study was the identification of significant knowledge gaps, which highlighted the impact of variables such as education, socioeconomic status, and healthcare accessibility. The study highlighted vital targeted interventions to enhance CVD awareness and knowledge, emphasizing preventive strategies and the empowerment of women. However, the study is not without limitations, including a constrained search scope of databases, potential language bias, a limited timeframe, supported only on existing literature without primary research, and an absence of detailed information regarding sample characteristics, which may potentially compromise the generalizability of the findings.

## Conclusions

In conclusion, this scoping review highlights the existence of significant differences in the awareness and knowledge about CVD among women in their reproductive age group. Lack of awareness regarding the symptoms and risk factors associated with CVD among women leads to delayed diagnosis and negative health outcomes. Factors such as limited education, low socioeconomic status, and the lack of access to healthcare facilities contribute to this knowledge gap. Moreover, young women, particularly those with APOs, have limited awareness and understanding of the risks related to CVD. It is essential to address these gaps in awareness and knowledge to enhance the health outcomes of women. Targeted interventions must be implemented to educate women regarding CVD risk factors and preventive measures before symptoms appear. To narrow the awareness gap, additional efforts should be made to lessen socioeconomic and educational disparities. By expanding awareness and knowledge, women can be empowered to engage in preventive measures and reduce their exposure to CVD development. Overall, this review emphasizes the significance of comprehensive education and awareness programs targeting women in their reproductive age group, with the aim of facilitating early detection, timely intervention, and improved management of CVDs.
